# Commentaries on Some of the Most Important Diseases of Children

**Published:** 1816-02

**Authors:** 


*Commentaries on some of the most important Diseases of Children.* By JOHN CLARKE, M. D. &c. &c. Part the First, Octavo, pp. 198. London, 1815.

THE work is dedicated to the Medical Students, who, in the last thirty years, have attended the author's lectures. We were among his earliest pupils, and we have now to delineate the work of our master, no longer a sojourner on this terrestrial scene! Alas, poor Yorick! he will no longer "set the table in a roar" with his inexhaustible fund of humour; but his writings will perpetuate his memory and his abilities. It is useless to deplore that fate which shortly awaits the pupil as well as the preceptor; the monarch as well as the subject! The *intellectual* labour, alone, like the soul, its source, survives the tomb, and outlives the pyramid!

By certain bills of mortality prefixed to the work, it appears that nearly a fourth of the human species, in the metropolis, die under two years of age; and of the survivors, about a fifth in the succeeding eight years. It is utterly inconsistent with the uniform goodness of the Creator, to suppose, that so many children are designed to be cut off at so early a period of their existence; and therefore, it must be attributed to some mismanagement. The severity of the winters, the vicissitudes of our atmosphere, and the improvident exposure of young children to cold, must lay the foundation of numerous diseases; while, in large cities, the tribe of contagious maladies has unusual force and means of reproduction. The morbid infirmities of infants have also been little studied, since, in all the works of the best writers, from Hippocrates downwards, scarcely any practical information on this subject is to be found. The reason is evident. Midwifery, and the treatment of children, till lately, have been committed to women and nurses, who were utterly incompetent to make, much less record, any useful observations. Since the time of Dr. William Hunter, many eminent characters have devoted themselves to the practice of midwifery and the study of infantile diseases; in consequence of which, much information was diffused by lectures, &c. through the minutest ramifications of medical society. Among the writers on these subjects (diseases of children) Dr. Armstrong, Dr. Harris, Dr. Underwood, Dr. Butter, Dr. Hamilton, and Dr. Burns, stand conspicuous; and, indeed, nearly complete the catalogue. Little confidence was placed in medical men, who almost wholly neglected the diseases of infants, and the charge of their disorders was given to ignorant old women and nurses, whose practice was confined almost exclusively to the promiscuous exhibition of rhubarb, magnesia, Castor oil, antimony, and opiates, under various forms.

"This last class of medicines has generally been exhibited (in very uncertain doses) in the forms of laudanum, syrup of white poppies, or under some empirical title, as Godfrey's cordial, or Dalby's carminative. These medicines have been ignorantly and indiscriminately given (in some instances, under the sanction of medical men) either because they did not themselves know what to do, or to fall in with the desires or prejudices of parents and friends. But the administration of this class of medicines requires the greatest skill in the physician. Nothing is more uncertain than the effects of opium on young subjects; and it ought never to be employed, even by medical men, except with the greatest caution, as it sometimes acts with much violence, and has proved deleterious in very small doses. Half a drachm of genuine syrup of poppies, and in some instances, a few drops of Dalby's carminative have proved fatal in the course of a few hours to very young infants." 33.

Dr. Clarke justly considers antimonial medicines as very unsafe in the diseases of children, and gives an instance where one quarter of a grain of tart. antim. destroyed a young child. Ipecacuanha is a safe emetic in this class of patients.

The disorders of infants having been represented as obscure, because they are incapable of describing their own complaints, Dr. C. directs our attention to the following particulars, which will be found indicative of health or disease. Their colour, figure, fatness, leanness, paleness, redness. The tone of the skin, the general or local heat, or coldness, gestures, cries, brightness or dullness of the eyes, the state of the pupil, the direction of the eyes, the state of the ossification, the straightness or deformity of the skeleton, the tone or atony of the muscular fibres, the state of the abdomen, the enlargement of the viscera, the state of the respiration, the various secretions, the excretions, their number, colour, consistence, texture, smell, &c. present themselves to the attentive practitioner, and leave him without an excuse, if he neglects his duty in the cure of infantile ailments.

The regulation of the food of children is an important prophylactic consideration. It is unnatural to suppose, that a child's mouth without teeth, and that of an adult furnished with the teeth of canivorous and graminivorous animals, are designed by the Creator for the same sort of food. If the mastication, and the admixture with saliva, of solid food, animal or vegetable, be necessary for digestion, then solid food must be improper for infants, where the power of mastication exists not. To give an infant the best chance of health, it should live exclusively upon the milk of its *mother*, or at least of a healthy nurse. If this cannot be done, the food should be entirely *fluid*, and taken by suction till it has teeth. Ass's milk is the best substitute for that of the mother; cow's milk is too rich, and is easily curdled by the gastric juice in the stomach. When ass's milk cannot be procured, Dr. C. recommends two parts of cow's milk skimmed, with one of gruel made from pearl barley, grits, rice, or arrow root. If this disagrees, weak mutton, chicken, or beef broth, clear and free from fat, mixed with any of the farinaceous decoctions abovementioned. As soon as the child has got any of the incisores, solid farinaceous matter, boiled in water, beaten through a sieve, and mixed with a small quantity of milk, may be employed, and then, for the first time, the child should be fed by the hand. When the molares protrude, the bread needs not be beaten through a sieve, because the child has now an apparatus for grinding it. Solid animal food should not be eaten till the child has all the cuspidati, or canine teeth, and then only once a-day. Water, the beverage of nature, is the proper drink of children.

### Dentition.

Where children have been *naturally* fed, an increased flow of saliva generally precedes and mitigates dentition. The explanation of this has been shewn in our review of Dr. Parry's work. Improper food, and too much of it, will render dentition difficult, from the plethoric state of the habit, or bad state of the digestive organs thereby induced. Too warm clothing about the head, and sleeping on soft pillows conduce to the same. The formation of the teeth naturally invites blood to the head, and by these erroneous measures, adopted to prevent cold, more blood is determined to that part, from which bad consequences arise. Those children whose heads are kept most cool, and are daily washed with cold water, are least liable to serious complaints at the time of teething. Weakly children, too, from being less disposed to inflammatory affections, go through the process of dentition more easily than those who are robust. A natural salivation and a natural diarrhœa, prevent or ameliorate the evils of teething; the former, by the local discharge from the inflamed parts; the latter, partly by determining the circulation to the intestines, but principally by diminishing the quantum of the circulating fluids, and the momentum of the blood to the head. We have no means of raising an artificial salivation in children, but can easily excite artificial diarrhœa. For this purpose, Dr. C. recommends the *saline* purgatives in preference to all others, and particularly the sulphate or supersulphate of potash.

When eruptions about the body, but especially the head, face, and ears, appear during dentition, if local remedies are employed to cure them, purgatives should never be omitted, otherwise their removal will frequently be followed by organic affection of the brain. Indeed, if these cutaneous diseases are not very important or troublesome, they are better left alone. If they must be healed, they should be healed slowly, and the most minute attention paid to the diet and state of the bowels. The same remark applies to the ophthalmia of children during dentition; many cases having fallen under Dr. Clarke's notice, where, from inattention to the above circumstances, inflammation of the brain has been produced, and ultimately effusion of water in the ventricles.

When the gums are much tumefied, they should be freely divided; the discharge of blood lessening the inflammation of the part, and the determination to the head. If the division of the gums be *unnecessarily* performed, no harm can arise; but great mischief may follow its omission, when necessary. It is not enough to scarify the gum, but the incision should be carried down to the tooth. When the molares are rising, *transverse* incisions should also be made to set the tooth quite at liberty. In children of full habits, the above directions are essentially necessary; and if the symptoms of irritation do not yield to purging and dividing the gums, the tepid bath, once or twice a day, and small doses of ipecacuanha, with saline draughts, will be found advantageous. Opium in every shape, is prejudicial in these cases, as it draws a veil between the physician and the disease. Fomentations, however, of decoct. anthem. with ex. pap. alb. (half an ounce to a quart) may be applied by means of a flat sponge to the side of the face, without the inconvenience of opiates internally. The approach of the cuspidati is usually attended with most mischief, for which it is difficult to assign a reason; probably it arises from greater irregularities in diet, before the age at which *these* teeth appear.

### CONVULSIONS.

This is a vague term, but our author confines it to "those convulsions, any single paroxysm of which agrees with the ordinary definition of epilepsy, and also to some other *spasmodic affections* not admitting of an accurate definition, yet arising from the same causes, and requiring similar treatment." Page 81. The most common specimen exhibits an universal spasmodic contraction of all the voluntary, and many of the involuntary muscles of the body, accompanied by foaming at the mouth, protrusion of the tongue, staring of the eyes, distortion of the eye-balls, laborious and obstructed respiration, sometimes accompanied with a violent redness of the face and scalp, at the beginning of the paroxysm, followed by a purple colour of the whole body, at the end of it. This last is sometimes a fatal sign. To the symptoms above enumerated, a state of coma and insensibility generally succeeds, and the child at last falls into a profound sleep, which often lasts for many hours. From this it awakes, in some instances, unimpaired in mental faculties, and without a repetition of the convulsions; but in general they recur, unless the cause be very temporary, and if not checked, too often prove fatal. Partial paralysis sometimes succeeds, and is slowly removed. In others, the mental faculties are destroyed, and the child grows up an ideot! Sometimes strabismus, chronic epilepsy, or chorea is the sequela. Dr. C. is of opinion, that convulsions are *never* an idiopathic disease, they will most frequently be found to have been preceded by febrile symptoms, drowsiness, yawning, sighing, increased irritability, impatience of sound and light, and by some derangement in the digestion or respiration.

Dr. Clarke here describes a *peculiar species of convulsion*, which is little known to practitioners. It occurs by paroxysms, the child being suddenly seized with a spasmodic inspiration, consisting of distinct attempts to fill the chest, between each of which, a squeaking noise is often made. The eyes stare; the face and extremities, if the paroxysm continue long, become purple; the head is thrown backward; the spine is often bent, as in opisthotonos; at length a strong expiration takes place; a fit of crying succeeds, and the exhausted infant falls asleep. In one of these a child sometimes, though not often expires. They occur often in the day, and are brought on by straining or fretting. They commonly take place after a full meal, or on waking from sleep. It is not croup, but altogether of a

*convulsive* character. It is frequently preceded or accompanied by a bending downwards of the toes: clenching of the fists : insertion of the thumbs into the palms of the hands, and bending the fingers upon them. This *last* often exists for a long time in children, without being observed, though it is an unfavourable symptom, and frequently the forerunner of convulsive disorders. Convulsions of this description are generally confined to the first three years of existence, and seldom occur where the child lives by sucking till the teeth protrude.

Dr. C. is of opinion, that in every kind of convulsions, whatever may be the remote cause, the brain is at the time, *organically* affected, either directly or indirectly. Directly, when they arise from phrenitis, hydrocephalus, suppressed eruptions, inflammation of the eyes, &c.; or when they appear on the accession of some cutaneous disease, as scarlet fever, small-pox, or measles. *Indirectly*, when occasioned by overloaded stomach, indigestion, peripneumony, inflammation or suppuration in the cavity of the pericardium, glandular or other tumours pressing on the large vessels leading to the lower extremities, infantile fever, marasmus.

"Many circumstances concur to prove, that even in chronic cases of *epilepsy*, both in children and in adults, some *pressure* on the brain is the *immediate* cause of a paroxysm, either from *inordinate fulness of the vessels*, &c. p. 91.

Our readers are now aware of the coincidence of opinion on this subject, between Dr. Parry and Dr Clarke. Of the manner in which the brain is *directly* affected in phrenitis, &c. by an increased momentum of blood to that organ, we need not speak; but the *indirect* pressure may occur, as has been abovementioned, from many causes, which have a tendency to prevent the free circulation thro' the rest of the body. Dr. C. mentions a recent case. where universal convulsions had succeeded to a long continuance of the *peculiar convulsive affection*, described above, and terminating in death. Dissection shewed fulness of the vessels of the brain, and water in the ventricles.

"The thorax was free from disease; but in the abdomen, a mass of enlarged mesenteric glands compressed the great vessels against the spine, *below the origin of the emulgent arteries*, so as to detain the blood in the upper parts of the body, and overcharge the vessels of the head." Page 95.

There was in this instance, an extraordinary secretion of urine, which is accounted for by the situation of the tumor.

In another instance of death from convulsions, the pericardium was found full of pus. The difficult transmission of the blood through the heart occasioned the venous turgescence in the brain.

### Treatment of Convulsions.

It is obvious, that some cases must be altogether beyond the power of medicine; such as those which depend upon scrofulous or other tumors, situated in the substance of the brain, dura mater, &c. or upon bony tumors arising from the inner table of the skull. Congenital hydrocephalus is of this kind, and perhaps many other derangements of structure not capable of being detected in our present state of physiologo-anatomical knowledge of the brain. It has been urged, that if convulsions are produced by permanent organic derangement, they should be more constant; but Dr. Parry and Dr. Clarke shew, that they are in these cases "immediately excited by some *increase* of pressure, occasioned by an *acceleration* of the circulation." 100. Thus it would be very difficult, during life, to form any idea that there existed any tumor pressing on the lower part of the aorta, and occasioning determination to the brain, as happened in the case above stated. Although the symptoms of hydrocephalus are well enough marked, when the head has increased in bulk, the sutures and fontanelles enlarged, the pupils become dilated, the senses blunted, and the mental faculties defective; yet, in early life, congenital hydrencephalus may exist, and betray no symptoms. The whole system is, as it were, adapted to the morbid structure of the brain, the ordinary functions sometimes proceeding naturally, and the child appearing to thrive. In other instances, only slight convulsive affections exhibit themselves, such as a propensity to clench the fist. In some, the digestion will be slightly impaired, with irregular and bad discharges from the bowels, and the disease will be neglected. At length, unequivocal symptoms of an oppressed brain occur, or actual inflammation is excited, and convulsions terminate the scene! Sometimes they are repeated from time to time, and the child drags on a miserable existence for months, or even years.

It is not often that convulsions preceding eruptive diseases prove fatal, because they are soon followed by a spontaneous determination to the skin, by which the blood is uniformly diffused over the whole body, and the circulation through the head proportionably lessened.

Convulsions occurring during dentition, the gums are always to be examined. If turgid, they are to be scarified; if inflamed, very freely so, to remove the whole tension of the gums, and of the membrane which covers the tooth. The lenity of the surgeon, on these occasions, is the greatest cruelty to the child. The bowels are to be freely opened, both by clysters and brisk cathartics, till the evacuations become copious. Calomel may be first given, and afterwards infus. sennæ with manna and tinct. jalap. in repeated doses, adapted to the age of the patient.

"It may be sufficient to observe," says Dr. C. "that children require much larger doses of purgative medicines, in proportion to their age, than adults. It is remarkably the case with this class of medicines; whilst it is found that they are more powerfully affected by opiates, and by preparations of antimony, so that the writer has known a very small quantity of syrup of poppy, and a very diluted solution of tartrite of antimony, both in a vinous and aqueous medicine, attended with deleterious consequences." 106.

The warm bath, from 92 to 94, should be employed, and, if necessary, repeated. Its utility is explicable on the principle of diffusing the circulation to the surface, and relieving the brain. For the latter purpose, blood is to be taken from the head by leeching, cupping, or opening the external jugular. No inconvenience can follow the loss of blood, even if *unnecessarily* taken away; but infinite mischief may result from the neglect of this measure. When, by the above means, the convulsions are not relieved, blisters are to be applied to the lower extremities.

As in phrenitis, both of children and adults, the application of cold fluids or ice to the shaven scalp has been attended with the best effects, so it is a very useful remedy in convulsions. The administration of opiates requires the utmost consideration and circumspection. They certainly should never be given, on any account, until it is clearly ascertained that there is no danger of pressure on the brain, or inflammation of that organ; and never until the bowels have been completely unloaded, lest the stupor arising from a compressed brain be mistaken for the effects of opium, and the time, when alone relief could have been given in inflammation of the brain, should be allowed to pass by, never to be recalled!

When, however, we are sure that no congestion or inflammation is going on in the head, opium in small doses, cautiously repeated, may be administered with advantage, as it will sometimes diminish pain, by lessening the sensibility and irritability of the patient. It is needless to caution the practitioner to keep the intestinal canal free during the exhibition of the opium. To shorten the duration of an individual paroxysm, there is scarcely any stimulus so powerful as the plentiful affusion of cold water over the face, the body being placed in a horizontal position, with the face upwards. This will frequently cut it short, when all other applications fail.

Next to this, the volatile ammonia, plentifully inhaled, will be found a very useful stimulus. Dr. C. thinks, that the advantage of this application arises from its occasioning a full expansion of the chest, and taking off the spasmodic affection of the muscles of inspiration which occasions the blue colour observable in patients during the continuance of the convulsion; a circumstance depending on the passage of the blood from the right to the left side of the heart, through the lungs, being impeded during the spasm of these muscles.

These are the only means which Dr. Clarke has ever seen of much advantage in convulsions. He puts no faith in the long list of antispasmodics, though recommended by many respectable practitioners. As popular prejudice, however, runs in favour of them, they may be employed with *safety,* though they should not supersede the more active remedies recommended above.

The two concluding and most important Chapters of the work shall form the subject of another article in our next Number. We hope that our analysis on this, as on other occasions, will be found to present—not the "*disjecta membra*" of works in motley patch-work, but a luminous and well-defined miniatnre of each.

